# Rapid functional diversification in the structurally conserved ELAV family of neuronal RNA binding proteins

**DOI:** 10.1186/1471-2164-9-392

**Published:** 2008-08-20

**Authors:** Marie-Laure Samson

**Affiliations:** 1 Laboratoire Signalisation, Développement et Cancer, UMR8080 Développement et Evolution, CNRS and Université Paris Sud, Bâtiment 442 bis, 91405, Orsay Cedex, France

## Abstract

**Background:**

The Drosophila gene *embryonic lethal abnormal visual system *(*elav*) is the prototype of a gene family present in all metazoans. Its members encode structurally conserved neuronal proteins with three RNA Recognition Motifs (RRM) but they paradoxically act at diverse levels of post-transcriptional regulation. In an attempt to understand the history of this family, we searched for orthologs in eleven completely sequenced genomes, including those of humans, *D. melanogaster *and *C. elegans*, for which cDNAs are available.

**Results:**

We analyzed 23 orthologs/paralogs of *elav*, and found evidence of gain/loss of gene copy number. For one set of genes, including *elav *itself, the coding sequences are free of introns and their products most resemble ELAV. The remaining genes show remarkable conservation of their exon organization, and their products most resemble FNE and RBP9, proteins encoded by the two *elav *paralogs of Drosophila. Remarkably, three of the conserved exon junctions are both close to structural elements, involved respectively in protein-RNA interactions and in the regulation of sub-cellular localization, and in the vicinity of diverse sequence variations.

**Conclusion:**

The data indicate that the essential *elav *gene of Drosophila is newly emerged, restricted to dipterans and of retrotransposed origin. We propose that the conserved exon junctions constitute potential sites for sequence/function modifications, and that RRM binding proteins, whose function relies upon plastic RNA-protein interactions, may have played an important role in brain evolution.

## Background

The *elav *(*embryonic lethal abnormal visual system*) gene of *D. melanogaster *was the the first identified member of a family of neuronal RNA binding proteins that is conserved in metazoans [1,2]. The proteins in this family contain three RNA Recognition Motifs (RRM), with a hinge region separating the second and third RRMs and an optional non-conserved N-terminal region. The hinge includes signals essential for nuclear export and subcellular localization [3].

RRM are common protein domains found in all life kingdoms. In humans, there are 497 genes encoding RRM containing proteins, which represent 2% of the human gene products. Proteins containing one or several of these domains are capable of interacting in a sequence specific manner with single stranded RNA molecules and of directing the assembly of multiprotein complexes [4,5]. In spite of the remarkable sequence conservation of the RRM domains, RRM-containing proteins perform numerous functions, intervening at all the possible steps of RNA metabolism. The RRM domain is composed of about 90 amino acids and contains a conserved octapeptide termed RNP-1 (ribonucleoprotein motif) and a conserved hexapeptide termed RNP-2. Structural studies indicate that four antiparallel beta-sheets form the RNA interaction surface, with RNP-1 and RNP-2 on the two inner sheets (beta 1 and beta 3). In RNA-RRM complexes, nucleotides establish contacts with residues in the RNPs, with regions in the RRM beyond the RNP domains also involved in RNA recognition. The plasticity of RRMs in their sequence-specific recognition of topologically diverse RNA is likely to be correlated with their presence in a variety of proteins involved in the diverse steps of post-transcriptional regulation.

There are three *elav*-related genes in *D. melanogaster*. The *elav *gene encodes a nuclear product present in all neurons throughout development and is required for the differentiation of postmitotic neurons and their maintenance [1]. The *rbp9 (RNA binding protein 9) *product is present in neuronal nuclei starting at the third larval instar and also in the cytoplasm of cystocytes during oogenesis. Although neuronal expression is predominant, *rbp9 *mutations reveal a role in cystocyte proliferation and differentiation, but no neuronal defects have been reported [6,7]. The expression of *fne (found in neurons) *resembles *elav*'s, but with a slightly delayed onset. FNE is cytoplasmic, but the *elav *and *fne *genes interact, suggesting protein shuttling [8,9]. The products of *elav *family members are essentially present in the nervous system, in all of the neurons in the case of *elav *itself, but more generally in subsets of neurons and/or neuroblasts and glial cells. Expression has also been detected in other tissues, in particular in testes and ovaries, or found to be ubiquitous (for instance [10]). Diverse molecular functions in the control of RNA half life, nuclear export, RNA 3' end formation, alternative RNA processing, polyadenylation and translation have been proposed for these proteins [9,11-17]. Multiple functions, both cytoplasmic and nuclear have been demonstrated for HuR, an ubiquitously expressed member of the human family [11,16,17].

The evolutionary relationship between members of the family are complex. For instance, the four human proteins share 74–91% identity, while the three Drosophila proteins share only 59–68% identity. The goal of the work reported here was to investigate these relationships. We found that the *elav *family has an eventful evolutionary history, somewhat masked by the high level of amino acid conservation of the gene products, but revealed by analysis of the gene structure of the different family members (11 species, 23 proteins). We attribute the rapid functional evolution of the family members, as opposed to the high level of sequence conservation, to the plasticity of the RRM domains, where small changes in critical positions have the potential to modify interactions with RNA.

## Results

### The paralogs *fne *and *rbp9 *share a conserved organization of their coding regions but *elav*, the third family member, is distinct

**Figure 1:**
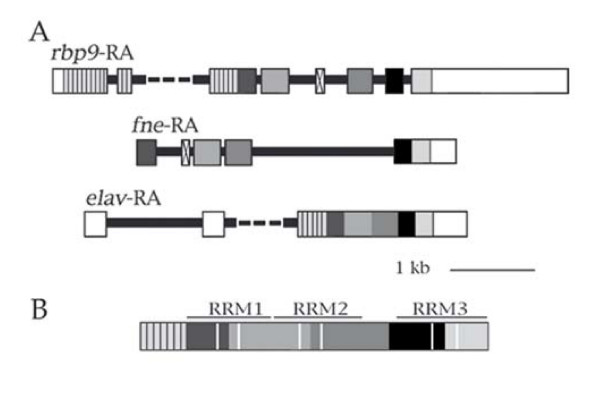
**Correspondance between exons and protein regions in the *elav *family of *D. melanogaster***. **A: RNA structures**. RNA nomenclature as in FlyBase, with details in the Methods. Boxes represent exons. The black horizontal lines are introns, with dashes respectively replacing the 5.8 kb long intron in the *rbp9-RA *transcript and the 2.2 kb long intron in the *elav-RA *transcript. White: non coding, Vertical stripes: non-conserved, Crossed: gene-specific mini-exons, respectively a 15 nucleotide long region present in alternative forms of *rbp9 *and a 45 nucleotide long region present in *fne*. All others are color coded based upon sequence similarity and according to exon-exon boundaries. **B: Schematic representation of the ELAV family protein products**. The color coding corresponds to that used for the RNA representation. The regions encoded by gene specific sequences have been omitted. RRM: RNA Recogntion Motif. The pairs of white vertical bars represent conserved motifs (RNP-1 and RNP-2) diagnostic of RRMs.

All three Drosophila paralogs *elav*, *rbp9 *and *fne *are essentially expressed in neurons. *elav *null mutants are embryonic lethal, while the *rbp9 *null mutation is viable, but surprisingly confers a female sterility phenotype. *fne *null mutants, although not fully characterized, are also viable (Zanini and Samson, in preparation). In order to understand the evolutionary mechanisms responsible for the generation of these paralogs, we examined their gene structure. Although their organizations are apparently quite distinct, we found remarkable conservation in the correspondance between exons and specific protein regions in *rbp9 *and *fne *(Fig. 1A). There are two differences (1) the presence of new mini-exons respectively specific for each of the two genes and (2) the use of a single exon in *fne *but two in *rbp9 *to encode the third RRM. Strikingly, this organization is totally different in the *elav *gene, whose complete ORF, except for the A of the ATG initiation codon, is encoded by a single exon.

### Conserved exon junctions are present in most *elav *orthologs

**Figure 2:**
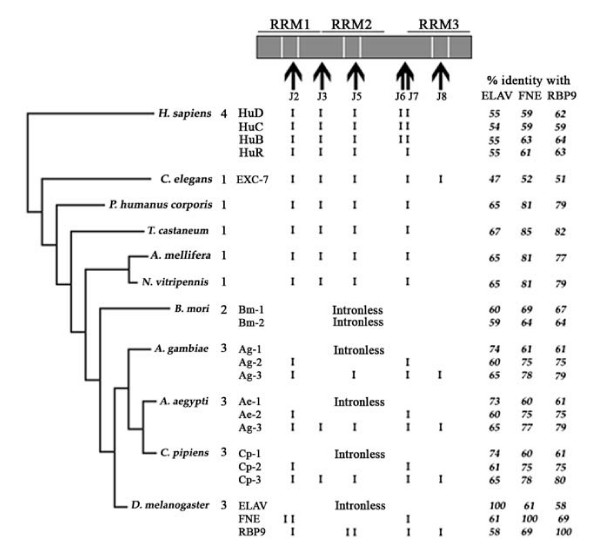
**Exon organisation of the *elav*-related genes in 11 metazoans**. The analyzed species are listed on the left, with classical phylogenetic relationships represented. The number of *elav*-like genes is listed next to the species names. Percentages of identity between their protein products and the *D. melanogaster *proteins ELAV, FNE and RBP9 are listed on the right side of the figure. At the top, a typical ELAV-like protein is represented, with its three RRMs and the hinge region between RRM2 and 3. The vertical arrows below point at protein regions that are, depending upon each of the 23 analyzed proteins, either encoded by exon-junctions (Jx, x = 1 to 8, see text) or by an internal exon sequence. The presence of the junction-encoded region is indicated by a vertical bar for each protein.

We took advantage of the recent sequencing of complete genomes [18-22] to survey the gene family in 11 species by (1) identifying all the family members and (2) comparing the organization of the ORF in exons. In humans, *D. melanogaster *and *C. elegans*, we extracted from the databases protein sequences deduced from cDNA analyses, and aligned genomic DNA with cDNA to determine the exon-intron structure. In other cases we used the predicted protein sequences, either published or computed for our purpose, as detailed in the Methods. We examined species from the chordata (1 species), arthropoda (9 species) and nematoda (1 species), for a total of 23 genes (Fig. 2).

First, we found that the size of *elav *families varies (one to four members) among the 11 species that we studied, with no clear relationship between family size and brain/animal complexity (Fig. 2). For instance, dipterans possess three *elav *genes, while the hymenopteran *Apis mellifora*, with ten times as many neurons as Drosophila, possesses only one gene. Levels of identity between the proteins encoded by the 23 genes are high, with the lowest score (47%) obtained in the comparison of *D. melanogaster *ELAV with the unique *C. elegans *protein. Between humans and Drosophila, there is 54–64% amino acid identity in the ELAV-related proteins, 38% identity for the arginase proteins (ubiquitous metabolic enzymes, see below) and 33% identity for the engrailed proteins (conserved transcription factors, not shown). The levels of ELAV-related protein identity are thus remarkable. The crystal structure of the first two RRM of human HuD associated with cfos RNA, identifies 12 amino acids whose side chain is making direct RNA contacts [23]. These residues are conserved in all 23 ELAV-like proteins that we examined, except for the arginine in RNP1 of the second RRM, which appears to be specific to the human proteins and to one of the *B. mori *ELAV-like, Bm-2. In the other species there is a conserved substitution by a lysine.

**Figure 3:**
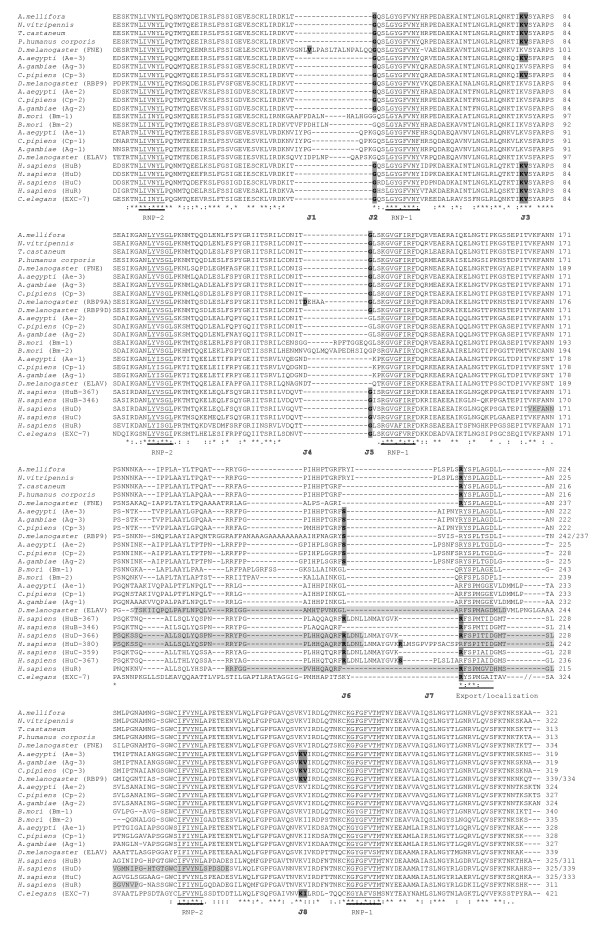
**Protein sequence comparison among 27 ELAV-like proteins forms**. Alternative protein forms are included, specifically for *Drosophila *RBP9 (A and D) and three of the human proteins (HuB, HuC and HuD, where HuX-n refers to the n amino acid long form of the HuX protein). "*" indicate that amino acids are identical in all 27 sequences, ":" and "." respectively indicate conserved and semi-conserved substitutions. The octamer RNP-1 and the hexamer RNP-2, diagnostic of RRMs, are underlined. Also underlined is a conserved octamer present in the region that is crucial for nuclear export and localization. The regions in light grey boxes have been mapped as necessary for these processes in *D. melanogaster *ELAV, human HuR and human HuD. We identified eight exon junctions labelled J1 to J8 (see text). Bold characters and dark grey boxes are used to identify amino acids encoded by exon junctions. When the splicing connects intact codons, two amino acids are bold (J3 and J8). The symbol//replaces 85 non-conserved amino acids in the *C. elegans *sequence.

Second, we found remarkable conservation of exon structure. From vertebrates to invertebrates, we identified eight exon junctions in the RRMs/hinge region (Fig. 3). We named them J1 to J8, from the most upstream to the most downstream. All are present in several phyla, except for J1 and J4 which are specific for FNE and RBP9 from Drosophila and are implicated in the generation of mini-exons in the sequence coding (alternative forms of) these proteins (Fig. 1 &3). Overall, the J2 junctions (respectively J3, J5 and J8, Fig. 2 &3) are unambiguously homologous since (1) the level of protein sequence conservation is such that the amino acid positions where the junctions intervene are clearly aligned and conserved (Fig. 3) and (2) nucleotide sequence analysis shows that at a given exon junction, the splice is at the same position in the codons: specifically between the first and the second bases of the spliced codons (for J2 and J5, as well as for the species-specific J1 and J4) or exactly between codons (J3 and J8). There are two exceptions to this strict conservation. First, J5 is interrupted in *rbp9 *of *D. melanogaster *by the intronic insertion of an alternative mini-exon, without alteration of the J5 5' or 3' splice sites. Second, in *fne*, J2 is split by the intronic insertion of a mini-exon, the J2 donor splice site is additionally shifted downstream while the J2 acceptor splice site is maintained (Fig. 3). Interestingly, the junctions J2 and J5 occupy the same position relative to RNP-1 in RRM1 and RRM2.

The junctions J6 and J7 map in a moderately conserved coding region, essential for nuclear export and proper subcellular localization (Reviewed in [2]), including only a conserved hexamer (R-SP----). Both J6 and J7 split the spliced codons between the second and the third bases. In this region, three types of events affecting the splicing seem to have occured independently: 1) the introduction of a mini-exon (in humans), that can be alternatively spliced (HuB), (2) the shift of the 5' splice site (example: *N. vitripennis *vs *T. castaneum*) (3) the shift of the 3' splice (example: the *T. castaneum *vs *Ae-2 *genes or the alternative human forms HuD-366 and HuD-380). Noticeably, the regions close respectively to J1/J2, J4/J5 and J6/J7 as well as the entire hinge region between RRM2 and RRM3 appear more variable than the rest of the protein.

### Intronless *elav*-like genes are present in Diptera and Lepidoptera

**Figure 4:**
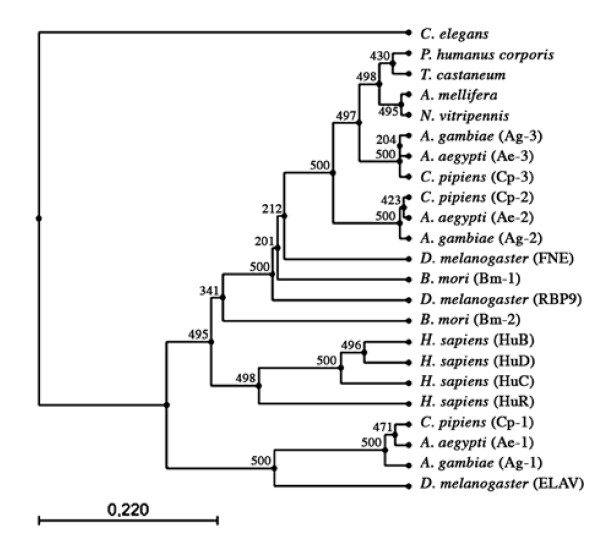
**Phylogenetic tree of 27 ELAV-like proteins**. Sequences were aligned and bootstrapped 500 times. Numbers near the branches are the bootstrap values, and the scale indicates the number of substitutions per site.

Interestingly, for six of the analyzed genes (*Ag-1*, *Ae-1*, *Cp-1*, *elav *in Diptera, and *Bm-1*, *Bm-2 *in Lepidoptera), the entire conserved region of the protein is encoded by a single exon. Based upon both their gene structure and the level of protein sequence identity, the dipteran intronless genes constitute a homogeneous *elav*-type group. In contrast, although intronless like *elav*, the *B. mori *genes encode proteins more similar to FNE/RBP9 than to ELAV. This observation suggests that distinct evolutionary forces shaped the *B. mori *genes and the dipteran *elav*-like intronless-genes, respectively. To evaluate this hypothesis, we performed a phylogenetic analysis of the 27 ELAV orthologs/paralogs, using the UPGMA algorithm, with bootstrap analysis (Fig. 4). This analysis shows with high confidence (bootstrap values greater or equal to 99%) that in dipterans, the proteins encoded by the intronless genes (*Ag-1*, *Ae-1*, *Cp-1*, *elav*) cluster together, while the two *B. mori *genes products cluster with the FNE/RBP9 sequences Similar results were obtained when performing sequence alignments using the neighbor joining method (not shown).

**Figure 5:**
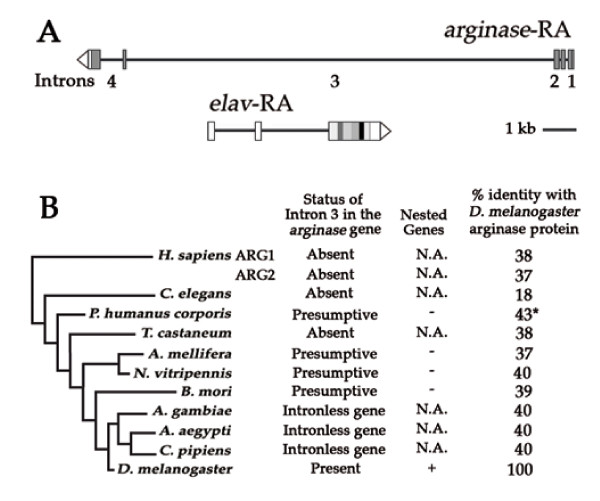
**A unique nested gene arrangement for the *elav *and *arginase *genes in *D. melanogaster***. A: The *elav *gene is nested inside the third intron of the *arginase *gene. Complementary strands are transcribed to generate the *elav *and *arg *RNAs with inverse polarities [28]. B: Examination of the relative *arg-elav *arrangement in 11 metazoans. There are two *arginase *genes in humans, only one in the other examined species. Column 1 documents the status of the *arginase *third intron. Column 2 specifies the nested (+) or independent (-) arrangement of the *arginase*/*elav *genes. N.A.: Not applicable. The third column indicates the percentage of amino-acid sequence identity of *D. melanogaster *compared with other species. *: N-terminally truncated arginase sequence for *P. humanus corporis*. See Additional file 3 for arginase alignments.

Because the *D. melanogaster elav *gene is nested in the third intron of the *arginase *gene [24], we probed the gene environment of the intronless *elav *orthologs that we report here. We found that the nested *elav*/*arg *gene organization is unique to Drosophila, specifically *D. melanogaster *and 11 additional Drosophila species whose genomes have recently been sequenced [25] (Fig. 5). In the 10 other non-Drosophila species examined here, there is no close linkage between the *arginase *gene(s) and the *elav *gene family members. In particular, the mosquitos, similar to *D. melanogaster*, each have three *elav*-like genes, including one intronless version, but unlike *D. melanogaster *they have an intronless *arg*, which obviously rules out the possibility of a nested gene. *In B. mori*, although the two intronless *Bm-1 *and *Bm-2 *genes map at loci distinct from the *arg *locus, an intron putatively homologous to the third intron of the *D. melanogaster arginase *gene is present (Fig. 5).

## Discussion

### The *D. melanogaster *gene *elav *is specific to the dipteran phylum and results from retrotransposition

The *elav *gene from Drosophila was the first identified member of this family, is considered as its prototype [1], and most of the subsequently discovered orthologs are named after it. However, the present analysis highlights unique characteristics of this gene that suggest it is of recent evolutionary origin, after the separation of dipterans and lepidopterans. Aside from *elav*, only the dipteran genes *Ae-1*, *Ag-1 *and *Cp-1 *encode proteins that are more similar to ELAV than to FNE and RBP9. In addition to the intronless *elav*-likes, dipteran genomes carry two genes encoding proteins of the type FNE/RBP9, also found in the seven other genomes analyzed. Thus *elav*, *Ae-1*, *Ag-1 *and *Cp-1 *represent a newly evolved gene form specific to dipterans.

In addition, the *elav *gene structure is suggestive of retrotransposition, a process considered significant in the evolution of genomes, including Drosophila [26]. The genes *Ae-1*, *Ag-1 *and *Cp*-1 from mosquitoes share with *elav *not only a higer level of similarity between their products, but also the property of having their ORF in a single exon. The absence of introns (restricted to dipterans and *B. mori *in this gene family) is atypical: we identified conserved exon junctions that are a landmark present in most of the *elav*-related genes. Furthermore, the *elav *gene of Drosophila is nested in the *arginase *gene. In humans, retrotransposition is an important contributor to the generation of nested genes [27]. We thus propose that *elav *originated from a recent retrotransposition event. It is possible that the same retrotransposition is at the origin of both the lepidopteran intronless *fne*/*rbp9*-like genes and the dipteran *elav*-like genes. A duplication of the retrotransposed gene in the ancestor to *B. mori *and different fates for the ancestral gene copies in the two groups would bring about the present situation. Alternatively, we do not exclude that independent retrotranspositions happened in lepidopteran and dipteran ancestral lineages.

Interestingly, the nested *arg*/*elav *arrangement found in *D. melanogaster *is not conserved in the mosquitoes, where the host gene (*arginase*) became intronless. This parallels the nested arrangement of the intronless *sina *gene in an intron of the *Rh4 *gene, as found in mosquitoes and nine species of the Drosophila genus. The remaining three species of the genus have an intronless *Rh4*, with a loss of the ancestral *Rh4 *copy where *sina *was originally embedded [28]. These situations show the lability of nested gene arrangements.

### *elav*: the genesis of a new function

It was unexpected to find that the copy number of *elav *family members varied from species to species. Given the maintenance of this gene family in all metazoans, we assume that there is a function for at least one, if not all, of the genes in each species. Mutants have been reported in only three species. The knockout of neuronal HuD in mice causes motor and sensory defects [29]. It is not excluded that the mild phenotype of this mutant is the consequence of gene redundancy. In *C. elegans*, cholinergic synaptic transmission is altered in mutants of the single *elav *ortholog EXC-7, which is expressed in a subset of neurons and other non-neuronal cells [30]. In both cases, viability and apparent morphology are normal. In *Drosophila melanogaster*, the vital gene *elav *is required in all neurons [1], whereas *rbp9 *is essential for female fertility [7] but does not affect viability. We recently generated null mutations of the *fne *gene (Zanini and Samson, in preparation), whose preliminary analysis indicates that they are viable in adults and lead to no apparent morphological defects. Aside from *elav *itself, characterized mutations of the *elav *gene family are viable, suggesting a non-vital function of the ancestral gene.

Considering that *elav *appears to be a new member of the family, its vital function is quite striking. This situation is reminescent of that of *Sex-lethal *(*Sxl*), a gene fundamental to sex determination in Drosophila, but which does not act as a sex determining factor in non-Drosophilids. The Drosophilid genomes indeed contain two *Sxl *paralogs (79% identity in *D. melanogaster*), while non-Drosophilids have one. It has been proposed that there was a duplication of the ancestral gene in Drosophilids and acquisition of a new function by one of the copies [31]. We believe that a retrotransposition of the *elav/fne/rbp9 *ancestor gene at the time of the separation of dipterans/lepidopterans led to a gene duplication and the evolution of a new function for *elav*.

### Conserved RNA binding proteins: a reservoir for accelerated functional evolution

We have pointed out that the ELAV-like proteins, including ELAV itself, have maintained a high level of sequence conservation between species, higher than that of engrailed, a conserved transcription factor with a homeodomain, or that of arginase, a ubiquitous metabolic enzyme that arose before the divergence of procaryotes and eucaryotes. This is intriguing in light of the extensively documented diversity of the properties of individual members of the family. First, although there is expression in the nervous system of at least one of the *elav *family members in every investigated metazoan (mammals, fishes, amphibians, birds, amphioxus, *C. elegans*, *D. melanogaster*), expression is also detected in other tissues and is even sometimes ubiquitous [2]. Second, the functions of these proteins are multiple, whether at the cellular level, where they include cell differentiation/survival [1,6,29,32] and cell proliferation/control of the cell cycle [7,33] or at the biological level, with impacts on motor/sensory activity, memory, fertility or viability [1,6,29,34]. Finally, the apparent subcellular localization of these proteins is diverse (nuclear, subnuclear, cytoplasmic or both), in agreement with diverse molecular functions [2,3].

The data thus reveal a diversification of the functions and of the specificity of expression of ELAV family members and implies a diversification of the interactions with other macromolecules, most evidently the RNAs whose metabolism is regulated by the RRM containing proteins. The DNA duplications and retrotranspositions that occured in the *elav *gene families constitute a starting point for the diversification of gene function. Changes in cell or tissue specificity of expression are often linked to modifications of non-translated regulatory regions. However, changes affecting the sub-cellular localization, known to be dependent upon the hinge region between RRM2 and RRM3, or changes in the interactions with proteins or RNA must depend upon the protein product of the *elav*-like genes.

Sequence alignments of the ELAV-like proteins shows that they are overall very conserved. But we were puzzeld by the fact some of the conserved exon junctions (J1/J2, J4/J5 and J6/J7) are adjacent to sequences that are among the most variable of the proteins. They include short insertions of amino acids, (alternative) exon addition and amino acid variations. The intron sequence indeed provides a potential source of sequence variability: it is conceivable that intron extremities become integrated into coding sequences by shifting of the exon boundaries. Alternatively, the intron can serve as the site of insertion of a new exon. An additional surprising point was the fact that these variable micro regions are almost directly upstream of important conserved motifs, specifically RNP-1 (in RRM1 and RRM2) and the octapeptide in the region essential for nuclear export and subcellular localization. The modification of residues outside of the RNP has the potential to alter the interactions between the RRM and an RNA [5]. Additionally, alterations of the region responsible for nuclear export/cellular localization modify this function (reviewed in [2]). We thus propose that the maintenance of the exon junctions is vital to the evolution of the ELAV family, in particular the generation of new functions. As a consequence, one would predict that RRM1, RRM2 and the hinge region have prominent roles in functional specificity. It may be significant in this respect that RRM3 replacements in ELAV by RRM3 from RBP9 or HUD are fully functional, while RRM1 or RRM2 replacements by corresponding RRMs from RBP9 or SXL are largely non-functional [35].

More generally, it seems that RRM-containing proteins could serve as favorable targets for the rapid evolution of gene functions. Because of the structural versatility of the RRM domain, it can be adapted for sequence specific recognition of many different nucleic acid structures and different protein partners [5]. The SXL protein, a crucial regulator of sex determination in Drosophila contains 2 RRM, and appears to be the result of such a rapid adaptation of function. In the search for genetic changes that distinguish our brains from that of our ancestors, the focus has been on the identification of non-synonymous changes in coding regions and the modification of regulatory sequences [36]. Our work suggests that the very conserved RRM-containing proteins may have contributed to human brain evolution, especially when considering the fundamental importance of the regulation of RNA metabolism in neurons, where alternative splicing [37] and localized RNA translation and degradation [38,39] take place with impacts on cortex development, neuronal regeneration and plasticity.

## Conclusion

The *elav *gene family encodes proteins with three RNA Recognition Motifs (RRM) acting as neuronal post-transcriptional regulators in all metazoans. Since they show remarkable sequence conservation, the documented diversity of their molecular roles is unexpected. We report the occurence of *elav*-like gene duplications and deletions in metazoans, and show that the vital *elav *gene of Drosophila is newly emerged, specific to dipterans and of retrotransposed origin, challenging its status of prototype for the family. These findings, together with the plasticity of the interactions between RRM and RNA, suggests that the *elav*-like proteins may have played an important role in the evolution of the gene functions crucial in brain evolution.

## Methods

### cDNA sequences used for the analysis of coding sequence organization in the *elav *gene family of *Drosophila melanogaster*

We used the transcripts data from FlyBase [18] to assess the relationship between RNA and protein coding regions. Multiple RNA isoforms from one gene were taken into account if they were a source of polypeptide diversity. For instance, seven alternative RNA forms have been reported for *rbp9*, which are predicted to encode six distinct polypeptides. Only one level of variation was relevant to the present analysis, that is the alternative inclusion of a mini-exon that causes the addition of 15 nucleotides (five amino acids), hence the choice of using the *rbp9-A *and the *rbp9-D *RNA forms, that differ by the presence/absence of the mini exon. In the case of both *fne *and *elav*, several transcripts have been reported but they encode a single polypeptide.

### Identification of *elav *orthologs in completely sequenced genomes and prediction of ELAV-like protein sequences

We used protein sequences from the data bases deduced from cDNA analysis whenever possible, with NCBI accession numbers as follows: in humans BAD92531 (HuB, 367 amino acids), AAH30692/Q12926-2 (HuB, 346 aa), AAA58677 (HuC, 359 AA), AAH14144/Q14576 (HuC, 367 AA), AAH36071/Q8IYD4 (HuD, 366 aa), AAK57541/AAK57541 (HuD, 380 aa), AAH03376/Q15717 (HuR, 326 aa), in *D. melanogaster *AAA28506 (ELAV, 483 aa), AAF43091 (FNE, 356 aa), AAF51179 (RBP9 isoform A, 647 aa) and AAN10401 (RBP9 isoform D, 642 aa), in *Caenorhabditis elegans *NP_496057 (EXC-7, 456 aa). UniProtKB/Swiss-Prot Accession numbers are also provided for further details on the proteins: Q12926 (HuB), Q14576 (HuC), Q8IYD4 (HuD), Q15717 (HuR), P16914 (ELAV,), Q9VYI0 (FNE), Q9VQJ0 (RBP9) and Q20084 (EXC-7).

When no cDNA sequences were available, we performed searches of the entire genomes using the tblastn program [40] to identify orthologs of ELAV-related genes. We analyzed the genomic regions encoding these orthologs by performing a three frame translation of the genomic sequences, and using the gene prediction program genescan [41] as well as a splice site prediction program [42]. The predicted protein coding sequences were the result of integration and manual review of these data.

Using the procedures detailed above to identify *elav *orthologs, we reviewed predicted protein sequences that have been proposed for *Apis mellifora*, *Aedes aegypti *and *Anopheles gambiae *[19]. Some of our conclusions were consistent with the automated predictions of genome projects (*A. mellifora*, XP_394166, 343 aa), but we edited sequences of *A. aegypti*, and *A. gambiae *ELAV orthologs. The decision of editing was based upon the identification of manifest errors in the automated predictions, such as the prediction of a four base pair intron 5'-CCCT-3', missing the consensus GT-AG sequences typically flanking introns for the Ag-3 predicted transcript (XM_309157). For those two species, as well as for those where no prediction had yet been proposed, we relied upon the above procedure to identify and propose predicted sequences of ELAV orthologs. They respectively derive from genomic sequences CH477489 (Ae-1), CH477672 (Ae-2), CH477401(Ae-3) in *A. aegypti*, from CM000357 (Ag-1), CM000360 (Ag-2), CM000359 (Ag-3) in *A. Gambiae*, DS231997 (Cp-1), DS232556 (Cp-2), DS231816 (Cp-3) in *Culex pipiens*, CM000276 in *Tribolium castaneum*, DS265619 in *Nasonia vitripennis*, AADK01020611 (Bm-1), CH391062 (Bm-2) in *Bombyx mori *and DS235033 in *Pediculus humanus corporis*.

In our analysis we used only the approximately 325 amino acids region of the proteins including the three RRM and a hinge region that links RRM2 and RRM3, because the N-terminus, when present, is not conserved. The sequences used are listed in Additional file 1.

### Identification of *arginase *genes in completely sequenced genomes and prediction of arginase protein sequences

Arginase sequences have been deduced from cDNA sequences for several species: human (ARG1: P05089, Arg2: P78540), *D. melanogaster *(Q9NHA5), *C. elegans *(Q22659). For the other species, we used the procedure described above to propose arginase sequences. The protein sequences derive from genomic sequences CH477248 in *A. aegypti*, from CM000359 in *A. gambiae*, DS232533 in *C. pipiens*, CM000280 in *T. castaneum*, DS265617 in *N. vitripennis*, CH389642 in *B. mori *and DS235286 in *P. humanus corporis*. We were not able to predict a complete *P. humanus corporis *arginase sequence, because of the lower level of conservation. See Additional file 2 for the arginase sequences.

### Protein sequence alignments and percentages of identity

Alignments were performed with the ClustalW program using default parameters [43]. In the case of arginases, we focused on the region homologous to that including intron 3 in *D. melanogaster*. The values for percentages of identity were extracted from the ClustalW score tables.

### Phylogenetic analysis

We used the CLC combined workbench (CLC bio A/S) version 3.6.2 to align the 27 protein sequences with an unweighted pair group method using arithmetic averages (UPGMA) and to evaluate the reliability of the inferred tree with a bootstrap analysis (500 replicates).

## Authors' contributions

The author takes full responsability for the work. She asked the question, devised the approach, performed it, analyzed the results and wrote the manuscript.

## Supplementary Material

Additional file 1 **Fasta sequences of the three RRMs and the hinge regions of ELAV-like proteins**. 27 Fasta sequences. Click here for file

Additional file 2 **Fasta sequences of the arginases**. 12 Fasta sequences. Click here for file

Additional file 3 **Protein sequence comparison among 12 arginases from 11 metazoans**. Arginase sequences alignment with legend. Click here for file
