# Supplementary material for: Rapid functional diversification in the structurally conserved ELAV family of neuronal RNA binding proteins
Source: BMC Genomics. 2008 Aug 20;9:392. doi: 10.1186/1471-2164-9-392 (PMC2529313; doi:10.1186/1471-2164-9-392)
Supplement: Additional file 1 — Fasta sequences of the three RRMs and the hinge regions of ELAV-like proteins. 27 Fasta sequences. [file 1471-2164-9-392-S1.pdf]

Fasta sequences of the three RRMs and the hinge regions of ELAV-like proteins

>APIS
EESKTNLIVNYLPQSMTQDEIRSLFSSIGEVESCKLIRDKLTGQSLGYGFVNYHRPEDAEKAINTLNGLRLQNKTIKVSYARPSSEAIKGANLYVSGLPKNMTQQDLE
NLFSPYGRIITSRILCDNITGLSKGVGFIRFDQRVEAERAIQELNGTIPKGSSEPITVKFANNPSNNNKAIPPLAYLTPQATRRYGGPIHHPTGRFRYIPLSPLSRYS
PLAGDLLANSMLPGNAMNGSGWCIFVYNLAPETEENVLWQLFGPFGAVQSVKVIRDLQTNKCKGFGFVTMTNYDEAVVAIQSLNGYTLGNRVLQVSFKTNKSKAA

>BMORI-1
EESKTNLIINYLPQSMTQEEIRSLFSSIGEVESCKLIRNKGAAFPDALNHALHGGGQSLGYAFVNYHRPEDAEKAIATLNGLRLQNKTIKVSYARPSSEAIKGANLYV
SGLPKTMTQSELERLFSPYGRIITSRILCENSGGRPFTGGEQGLSKGVGFIRFDQRVEAERAIQELNGTIPKGASEPITVKFANNPSNNGKALAPLAAYLPAALRFPA
PLGRFSSGKSLLAINKGLQRYSPLAGELLGGVLPGAVGSEWCIFVYNLAPETEENVLWQLFGPFGAVQSVKVIRDLQTNKCKGYGFITMTNYDEAVVAIQSLNGYTLG
NRVLQVSFKTNKIKTI

>BMORI-2
NESKTNLIINYLPQTMTQEEIRSLFSSVGEVESCKLIRDKVTVFPDHILNGQSLGYAFVNYHRAEDAEKAVNTLNGLRLQNKIIKVSYARPSSDAIKGANLYVSGLPK
HMTQQELEKLFGPYGTIISSRILHENMNVGQLMQVAPEDHSIQGPSRGVAFIRYDQRCEAEAAIRELNGTIPPGGTTPMTVKCANNPSNQNKVLAPLTAYLAPTSTRR
IITPAVKALLAINKGLQRFSPLSDPLIQGNALGGSGWCIFVYNIGADTEESVLWQLFGPFGAVQSVKIIRDPSTNKCKGYGFVTMTNYDEAVVAIQSLNGYSLNGQVL
QVSFKTNKSKS

>TRIBOLIUM
EESKTNLIVNYLPQTMTQEEIRSLFSSIGEVESCKLIRDKVTGQSLGYGFVNYHRPEDAEKAINTLNGLRLQNKTIKVSYARPSSEAIKGANLYVSGLPKNMTQQDLE
SLFSPYGRIITSRILCDNITGLSKGVGFIRFDQRLEAERAIQELNGTIPKGSTEPITVKFANNPSNNNKAIPPLAAYLTPQATRRFAGPIHHPTGRFRYSPLAGDLLA
NSMLPGNAMNGSGWCIFVYNLAPETEENVLWQLFGPFGAVQSVKVIRDLQTNKCKGFGFVTMTNYDEAVVAIQSLNGYTLGNRVLQVSFKTNKSKTT

>NEMATODE
GESKTNLIINYLPQGMTQEEVRSLFTSIGEIESCKLVRDKVTGQSLGYGFVNYVREEDALRAVSSFNGLRLQNKTIKVSYARPSNDQIKGSNLYVSGIPKSMTLHELE
SIFRPFGQIITSRILSDNVTGLSKGVGFVRFDKKDEADVAIKTLNGSIPSGCSEQITVKFANNPASNNPKGLLSDLEAVQQAATTLVPLSTILGAPTLRATAGGIGPM
HHAPITSKYRYSPMGAITAVSQPTATLPADYLTTSALLQMSQLNALAGLNPFATATAVPDFTASLIAHQQQQHAVAQQHAAQTASPPATNGQVAGLAAHAQLSALSAS
VAATLPPSDTAGYCLFVYNLSSDTDDTLLWQLFSQFGAIVNVKILRDLTQQCKGYAFVSMSNYTEAYNAMLSLNGTNLAGKTLQVVFKSSTPYRA

>HUB-367
EDSKTNLIVNYLPQNMTQEELKSLFGSIGEIESCKLVRDKITGQSLGYGFVNYIDPKDAEKAINTLNGLRLQTKTIKVSYARPSSASIRDANLYVSGLPKTMTQKELE
QLFSQYGRIITSRILVDQVTGISRGVGFIRFDKRIEAEEAIKGLNGQKPPGATEPITVKFANNPSQKTNQAILSQLYQSPNRRYPGPLAQQAQRFRLDNLLNMAYGVK
RFSPMTIDGMTSLAGINIPGHPGTGWCIFVYNLAPDADESILWQMFGPFGAVTNVKVIRDFNTNKCKGFGFVTMTNYDEAAMAIASLNGYRLGDRVLQVSFKTNKTHK
A

>HUB-346
EDSKTNLIVNYLPQNMTQEELKSLFGSIGEIESCKLVRDKITGQSLGYGFVNYIDPKDAEKAINTLNGLRLQTKTIKVSYARPSSASIRDANLYVSGLPKTMTQKELE
QLFSQYGRIITSRILVDQVTGISRGVGFIRFDKRIEAEEAIKGLNQKPPGATEPITVKFANNPSQKTNQAILSQLYQSPNRRYPGPLAQQAQRFRFSPMTIDGMTSLA
GINIPGHPGTGWCIFVYNLAPDADESILWQMFGPFGAVTNVKVIRDFNTNKCKGFGFVTMTNYDEAAMAIASLNGYRLGDRVLQVSFKTNKTHKA

>HUC-359
DDSKTNLIVNYLPQNMTQDEFKSLFGSIGDIESCKLVRDKITGRDLGYGFVNYPDPNDADKAINTLNGLKLQTKTIKVSYARPSSASIRDANLYVSGLPKTMSQKEME
QLFSQYGRIITSRILVDQVTGVSRGVGFIRFDKRIEAEEAIKGLNGQKPLGAAEPITVKFANNPSQKTGQALLTHLYQSSARRYAGPLHHQTQRFRLDNLLNMAYGVK
RFSPIAIDGMSGLAGVGLSGGAAGGWCIFVYNLSPEADESVLWQLFGPFGAVTNVKVIRDFTTNKCKGFGFVTMTNYDEAAMAIASLNGYRLAERVLQVSFKTSKQHK
A

>HUC-367
DDSKTNLIVNYLPQNMTQDEFKSLFGSIGDIESCKLVRDKITGQSLGYGFVNYSDPNDADKAINTLNGLKLQTKTIKVSYARPSSASIRDANLYVSGLPKTMSQKEME
QLFSQYGRIITSRILVDQVTGVSRGVGFIRFDKRIEAEEAIKGLNGQKPLGAAEPITVKFANNPSQKTGQALLTHLYQSSARRYAGPLHHQTQRFRLDNLLNMAYGVK
SPLSLIARFSPIAIDGMSGLAGVGLSGGAAGAGWCIFVYNLSPEADESVLWQLFGPFGAVTNVKVIRDFTTNKCKGFGFVTMTNYDEAAMAIASLNGYRLGERVLQVS
FKTSKQHKA

>HUD-366
DDSKTNLIVNYLPQNMTQEEFRSLFGSIGEIESCKLVRDKITGQSLGYGFVNYIDPKDAEKAINTLNGLRLQTKTIKVSYARPSSASIRDANLYVSGLPKTMTQKELE
QLFSQYGRIITSRILVGQVTGVSRGVGFIRFDKRIEAEEAIKGLNGQKPSGATEPITVKFANNPSQKSSQALLSQLYQSPNRRYPGPLHHQAQRFRLDNLLNMAYGVK
RFSPITIDGMTSLVGMNIPGHTGTGWCIFVYNLSPDSDESVLWQLFGPFGAVNNVKVIRDFNTNKCKGFGFVTMTNYDEAAMAITSLNGYRLGDRVLQVSFKTNKAHK
S

>HUD-380
DDSKTNLIVNYLPQNMTQEEFRSLFGSIGEIESCKLVRDKITGQSLGYGFVNYIDPKDAEKAINTLNGLRLQTKTIKVSYARPSSASIRDANLYVSGLPKTMTQKELE
QLFSQYGRIITSRILVDQVTGVSRGVGFIRFDKRIEAEEAIKGLNGQKPSGATEPITVKFANNPSQKSSQALLSQLYQSPNRRYPGPLHHQAQRFRLDNLLNMAYGVK
RLMSGPVPPSACSPRFSPITIDGMTSLVGMNIPGHTGTGWCIFVYNLSPDSDESVLWQLFGPFGAVNNVKVIRDFNTNKCKGFGFVTMTNYDEAAMAIASLNGYRLGD
RVLQVSFKTNKAHKS

>HUR-326
DIGRTNLIVNYLPQNMTQDELRSLFSSIGEVESAKLIRDKVAGHSLGYGFVNYVTAKDAERAINTLNGLRLQSKTIKVSYARPSSEVIKDANLYISGLPRTMTQKDVE
DMFSRFGRIINSRVLVDQTTGLSRGVAFIRFDKRSEAEEAITSFNGHKPPGSSEPITVKFAANPNQNKNVALLSQLYHSPARRFGGPVHHQAQRFRFSPMGVDHMSGL
SGVNVPGNASSGWCIFIYNLGQDADEGILWQMFGPFGAVTNVKVIRDFNTNKCKGFGFVTMTNYEEAAMAIASLNGYRLGDKILQVSFKTNKSHK

>DMELAV
TETRTNLIVNYLPQTMTEDEIRSLFSSVGEIESVKLIRDKSQVYIDPLNPQAPSKGQSLGYGFVNYVRPQDAEQAVNVLNGLRLQNKTIKVSFARPSSDAIKGANLYV
SGLPKTMTQQELEAIFAPFGAIITSRILQNAGNDTQTKGVGFIRFDKREEATRAIIALNGTTPSSCTDPIVVKFSNTPGSTSKIIQPQLPAFLNPQLVRRIGGAMHTP
VNKGLARFSPMAGDMLDVMLPNGLGAAAAAATTLASGPGGAYPIFIYNLAPETEEAALWQLFGPFGAVQSVKIVKDPTTNQCKGYGFVSMTNYDEAAMAIRALNGYTM
GNRVLQVSFKTNKAK

>DMFNE
DESRTNLIVNYLPQTMTQEEMRSLFSSIGELESCKLVRDKVSGNLVLPASLTALNPALQQGQSLGYGFVNYVAEDAEKAVNTLNGLRLQNKVIKVSYARPSSESIKGA
NLYVSGLPKNLSQPDLEGMFASFGKIITSRILCDNISGLSKGVGFIRFDQRNEAERAIQELNGKTPKGYAEPITVKFANNPSNSAKAQIAPPLTAYLTPQAAAATRRL
AGALPSAGRIRYSPLAGDLLANSILPGNAMTGSGWCIFVYNLAPETEENVLWQLFGPFGAVQSVKVIRDLQTSKCKGFGFVTMTNYDEAVVAIQSLNGYTLGNRVLQV
SFKTNKTKTT

>DMRBP9
PDPKTNLIVNYLPQTMSQDEIRSLFVSFGEVESCKLIRDKVTGQSLGYGFVNYVKQEDAEKAINALNGLRLQNKTIKVSIARPSSESIKGANLYVSGLPKNMTQSDLE
SLFSPYGKIITSRILCDNITDEHAAGLSKGVGFIRFDQRFEADRAIKELNGTTPKNSTEPITVKFANNPSSNKNSMQPLAAYIAPQNTRGGRAFPANAAAGAAAAAAA
AAIHPNAGRYSSVISRYSPLTSDLITNGMIQGNTIASSGWCIFVYNLAPDTEENVLWQLFGPFGAVQSVKVIRDLQSNKCKGFGFVTMTNYEEAVLAIQSLNGYTLGN
RVLQVSFKTNKNKQT

>AE1
ETARTNLIVNYLPQTMTEEEIRSLFSSVGEVESVKLVRDKNVIYPGQPKGQSLGYGFVNFHRSQDAEQAVNVLNGLRLQNKVLKVSFARPSSEGIKGANLYISGLPKT
ITQEELETIFRPYGEIITSRVLVQEGNDKPKGVGFIRFDQRKEAERAIAALNGTTPKGLTDPITVKFSNTPGQNTAAKIVQPALPTFLNPQLTRRLGAIHHPINKGLA
RFSPMGGEVLDMMLPTAPTTGIGAIAPSGGWSIFIYNLAPETEENTLWQLFGPFGAVQNVKVIKDSATNQCKGYGFVTMTNYEEAMLAIRSLNGYTLGQRVLQVSFKT
NKAK

>AE2
EDSKTNLIVNYLPQTMTQEEVKSLFSSIGDVESCKLIRDKVTGQSLGYGFVNYHRAEDADKAINTFNGLRLQNKTIKVSFARPSSDAIKGANLYVSGLSKSMTQQDLE
ALFQPYGQIITSRILCDNITGLSKGVGFIRFDQRSEAERAIQQLNGTTPKGASEPITVKFANNPSNNINKAIPPLAAYLTPTPNLRRFPPGPIHPLSGRFSLPSNFSR
YSPLTGDLGTSVLSANAINGSGWCIFVYNLAPETEENVLWQLFGPFGAVQSVKVIKDLQTNKCKGFGFVTMTNYDEAVVAVQSLNGYTLGNRVLQVSFKTNNTKSKTN

>AE3
EDSKTNLIVNYLPQQMTQEEIRSLFSSIGEVESCKLIRDKVTGQSLGYGFVNYQRVEDASKAINTLNGLRLQNKQIKVSFARPSSEAIKGANLYVSGLPKNMLQADLE
SLFSPYGRIITSRILCDNITGLSKGVGFIRFDQRVEAEKAIKELNGTIPKGSTEPITVKFANNPSNTKTVPPLAAYLGPQAARRFPGPIHHPTGRFSAIPNYRYSPLA
GDLLANTMIPTNAIANGSGWCIFVYNLAPETEENVLWQLFGPFGAVQSVKVIKDLQTNKCKGFGFVTMTNYDEAVVAIQSLNGYTLGNRVLQVSFKTNKSKNS

>AG1
NNSRTNLIVNYLPQTMTEEEIRSLFSSVGEVESVKLVRDKNVIYPGQPKGQSLGYGFVNYHRPQDAEQAVNVLNGLRLQNKVLKVSFARPSSEGIKGANLYISGLPKT
ITQEELETIFRPYGEIITSRVLIQDGNDKPKGVGFIRFDQRKEAERAIQALNGTTPKGLTDPITVKFSNTPGQNAAAKVVQPALPAFLNPQLTRRLGAIHHPINKGLA
RFSPMGGEVLDMMLPAAPANGLNVAPSGGWSIFIYNLAPETEENTLWQLFGPFGAVQNVKVIKDAATNQCKGYGFVTMTNYEEAMLAIRSLNGYTLGQRVLQVSFKTN
KSK

>AG2
EDSKTNLIVNYLPQTMTQEEVKSLFSSIGDVESCKLIRDKVTGQSLGYGFVNYHRPEDAEKAINTFNGLRLQNKTIKVSFARPSSDAIKGANLYVSGLSKSMTQQDLE
NLFNAYGQIITSRILCDNITGLSKGVGFIRFDQRSEAERAIQQLNGTTPKGASEPITVKFANNPSNNINKAIPPLAAYLTPTPNLRRFPPGPIHPLSGRFSLPSNFSR
YSPLTGDLGSSVLSANAINGSGWCIFVYNLAPETEENVLWQLFGPFGAVQSVKVIKDLQTNKCKGFGFVTMTNYDEAVVAVQSLNGYTLGNRVLQVSFKTNNTKSKAN

>AG3
EDSKTNLIVNYLPQQMTQEEIRSLFSSIGEVESCKLIRDKVTGQSLGYGFVNYQRAEDASKAINTLNGLRLQNKQIKVSFARPSSEAIKGANLYVSGLPKNMLQADLE
ALFSPYGRIITSRILCDNITGLSKGVGFIRFDQRMEAEKAIKELNGTVPKGSTEPITVKFANNPSSTKTVPPLAAYLGPQAARRFPGPIHHPTGRFSAIPNYRYSPLA
GDLLANSMIPTNAIANGSGWCIFVYNLAPETEENVLWQLFGPFGAVQSVKVIKDLQTNKCKGFGFVTMTNYDEAVVAIQSLNGYTLGNRVLQVSFKTNKSKNA

>CP1
DNARTNLIVNYLPQTMTEEEIRSLFSSVGEVESVKLVRDKNVIYPGQPKGQSLGYGFVNFHRSQDAEQAVNVLNGLRLQNKVLKVSFARPSSEGIKGANLYISGLPKT
ITQEELEIIFRPYGEIITSRVLVQDGNDKPKGVGFIRFDQRKEAERAIAALNGTTPKGLTDPITVKFSNTPGQNSTAKIVQPALPTFLNPQLTRRLGAIHHPINKGLA
RFSPMGGEVLDMMLPTAPTNGLGAVAPSGGWSIFIYNLAPETEENTLWQLFGPFGAVQNVKIIKDSATNQCKGYGFVTMTNYEEAMLAIRSLNGYTLGQRVLQVSFKT
NKSK

>CP2
EDSKTNLIVNYLPQTMTQEEVKSLFSSIGDVESCKLIRDKVTGQSLGYGFVNYHRPEDAEKAINTFNGLRLQNKTIKVSFARPSSDAIKGANLYVSGLSKSMTQQDLE
ALFQPYGQIITSRILCDNITGLSKGVGFIRFDQRSEAERAIQQLNGTTPKGASEPITVKFANNPSNNINKAIPPLAAYLTPTPNLRRFPPGPIHPLGGRFSLPSNFSR
YSPLTGDLGTSVLSANAINGSGWCIFVYNLAPETEENVLWQLFGPFGAVQSVKVIKDLQTNKCKGFGFVTMTNYDEAVVAVQSLNGYTLGNRVLQVSFKTNNTKSKTS

>CP3
EDSKTNLIVNYLPQQMTQEEIRSLFSSIGEVESCKLIRDKVTGQSLGYGFVNYQRAEDASKAINTLNGLRLQNKQIKVSFARPSSEAIKGANLYVSGLPKNMLQSDLE
SLFSPYGRIITSRILCDNITGLSKGVGFIRFDQRTEAEKAIKELNGTIPKGSTEPITVKFANNPSNTKTVPPLAAYLGPQAARRFAGPMHHPTGRFSAIPNYRYSPLA
GDLLANSMIPTNAIANGSGWCIFVYNLAPETEENVLWQLFGPFGAVQSVKVIKDLQTNKCKGFGFVTMTNYDEAVVAIQSLNGYTLGNRVLQVSFKTNKSKNA

>NASONIA
EESKTNLIVNYLPQTMTQEEIRSLFSSIGEVESCKLIRDKLTGQSLGYGFVNYHRPEDAEKAINTLNGLRLQNKTIKVSYARPSSEAIKGANLYVSGLPKNMTQQDLE
NLFSPYGRIITSRILCDNITGLSKGVGFIRFDQRVEAERAIQELNGTIPKGSTEPITVKFANNPSNNNKAIPPLAAYLTPQATRRFGGPIHHPTGRFRYIPLSPLSRY
SPLAGDLLANSMLPGNAMNGSGWCIFVYNLAPETEENVLWQLFGPFGAVQSVKVIRDLQTNKCKGFGFVTMTNYEEAVVAIQSLNGYTLGNRVLQVSFKTNKSKAA

>PHC
EESKTNLIVNYLPQTMTQEEIRSLFSSIGEVESCKLIRDKVTGQSLGYGFVNYQRPEDAEKAINTLNGLRLQNKTIKVSFARPSSESIKGANLYVSGLPKNMTQQDLE
SLFSPYGRIITSRILCDNITGLSKGVGFIRFDQRMEAERAISELNGSIPKNSTDPITVKFANNPSNNNKAIPPLAAYLAPQAARRFAGPIHHPTGRFRYSPLAGDLLA
NSMLPGNAMNGSGWCIFVYNLAPETEENVLWQLFGPFGAVQSVKVIRDLQTNKCKGFGFVTMTNYDEAVVAIQSLNGYTLGNRVLQVSFKTNKTKTA
