# Supplementary material for: Rapid functional diversification in the structurally conserved ELAV family of neuronal RNA binding proteins
Source: BMC Genomics. 2008 Aug 20;9:392. doi: 10.1186/1471-2164-9-392 (PMC2529313; doi:10.1186/1471-2164-9-392)
Supplement: Additional file 2 — Fasta sequences of the arginases. 12 Fasta sequences. [file 1471-2164-9-392-S2.pdf]

Fasta sequences of the arginases:

>DMELANOGASTERARG
WSRKFASRSLRLHRLKSTGSTAPREPEQSLGIIGVPFAKGQAKQGVELAPDLLRQSSLRQVLQSSHDGLVIRDYGNLQYAVDEPLLQQQRVHYHHIRNYADFMACNRA
LIEQVKLMLVENTQFLAIGGDHAIGFGSVAGHLQHTPNLSLVWIDAHADINLHSTSQSGNIHGMPVSFLLEQLRNTWQHAGLQEIAPNCLPKDQLVYIGLRDIDPYEA
FILNKVGIRYYAMDTIDRVGVPKIIEMTLDALNPQNKIHVSFDIDALDSNVAPSTGTAVRGGLTLREGISIVEALRDTKRVQGVDLVEINPKLGSERDVRTTVESGLE
ILKSMFGYRRSGRWSNIDTGILGSD

>CULEXPARG
MLLLSKSSLVKLLVQRVGQANLSTVAGLEKFKKINYEKIGIVGVPFEKGQRKKGVGLGPKAIREAGLIDSIQEISNKLDIRDYGDIRYEALNLQGRLAANMKKLEHVA
SCTRFLSERVTQVLNEERLCLTLGGDHAIAIGSIDGHLKHCSDVAVIWVDAHADLNTNSTSPSGNIHGMPVALLAKELAYWPYIPGMDWQEPIISIKNMVYIGLRSVD
PYERLIIEKFGIHAFGMREVEQYGIHEVMRMALERVDPEGKKSLHVSYDIDSLDVLEAPSTGTSVRGGLTLREGIYIMEEAYNTGRLAAVDLVEVNPSIGTPDDVRKT
LDAAIHLLVAACGHNRRGNFADTLDIVGKKK

>AEDESAARG
MLRGKHLAKLLQNGSRHLSTDGLEKFRKINYEKIGIVGVPFEKGQRKKGVGLGPKAIREAGLIDSIQEISTKLDIRDYGDIQYEALNLQGRLATNMKQLEHVASCTKL
LSQRVTRVLNEDRLCLTLGGDHAIAIGSIDGHLKHCSDVGVIWVDAHADLNTNSTSPSGNIHGMPVALLAKELADYWPYIPGMDWQEPIISIKNMVYIGLRSVDPYER
VIIEKFGIHAFGMREVEKYGINDVMKMALERIDPEGKKSLHVSYDIDSLDVLEAPSTGTGVRGGLTLREGIYIMEEAYNTGRLAAVDLVEVNPSVGTPEDVRKTLDAA
IHLLVAACGHSRIGDIADTIDLIKK

>ANOPHELESGARG
MLPRIALARLVGRTGVTFGASTSSYCTSTADHSFATIKPKKINYERIGIVGVPFDKGQRKKGVGLGPKAIREAGLIDHIQEISPKLNIKDYGDIQYEALNFQGRKVGN
MKKLEHVASCSRNLSHQVTEVLDDDRLCITLGGDHAIAIGSIDGHLHHSKDVAVIWVDAHADLNTNSTSPSGNIHGMPVALLARELCDYWPYIPGMDWQEPIISIKNL
AYIGLRSVDPYERAIIEKFGINAFGMREVEKYGIREVMRMALERIDPNGERSLHVSYDIDSLDVLEAPSTGTSVRGGLTLREGIYIMEEAYGTGRLAAVDLVEVNPAI
GTPEDVRRTVEAAIHLLVAACGHSRKGDIADTLDLIQK

>NASONIAVARG
MLARTVKHFFRKIGERQYSKVGIIGVPFDKGQRKAGVGEAPDIIRSAGLVNELQGLGLNVHDYGNVQYETASINVDNMPNLGDVAACTQKLSEMVQQSLRDGRRVLTI
GGDHSIGIGSIDGHVKVDKDVAILWIDAHADLNTNKTSESGNVHGMPVALLASELADYWPYLPGMDWQQPIVSIRNVGYIGLRSVDRYERLVIEKFGITAFGMEDVER
FGIHEVVNMALRKIDPDNNRSLHVSFDIDSLDPLEAPSTGTPVRGGLSLREGIHLMEDLYRTNRLNALDLVEVNPRIGDKKSVDFTVEAAIHIIQAGFGYSRRGLKVP
EGITDLPLQTFR

>APISMEARG
MNSLRKIQSVIIRLGNRYYKKLGIIGVPFEKGQKGVAQGPEAIRKAGLMKELELLGLDVKDYGNILYKAKNVAEVDNMTHLGDVAGCTSKLSEQFQQILKKDRRILTL
GGDHSGIGTIDGHVKEKGDIALIWVDAHADLNTNKTSTTGLFHGMPVALLTSELADYWPHLPGMDWQKPMLSIRNVAYIGLREVDSYERLVIEKFGITAFGMEDIERY
GIHDVTYMALSKIDPNDSRSIHVSFDIDSLDPLEAPCTGTPVRGGLSLREAIHLMEVLYRTKRLNALDIVEINPYIGNKYDVQLTIGAAIHIQAGFGYSRRGLRVPEG
VTDIPLPTVK

>HUMANARG2
MSLRGSLSRLLQTRVHSILKKSVHSVAVIGAPFSQGQKRKGVEHGPAAIREAGLMKRLSSLGCHLKDFGDLSFTPVPKDDLYNNLIVNPRSVGLANQELAEVVSRAVS
DGYSCVTLGGDHSLAIGTISGHARHCPDLCVVWVDAHADINTPLTTSSGNLHGQPVSFLLRELQDKVPQLPGFSWIKPCISSASIVYIGLRDVDPPEHFILKNYDIQY
FSMRDIDRLGIQKVMERTFDLLIGKRQRPIHLSFDIDAFDPTLAPATGTPVVGGLTYREGMYIAEEIHNTGLLSALDLVEVNPQLATSEEEAKTTANLAVDVIASSFG
QTREGGHIVYDQLPTPSSPDESENQARVRI

>HUMANARG1
MSAKSRTIGIIGAPFSKGQPRGGVEEGPTVLRKAGLLEKLKEQECDVKDYGDLPFADIPNDSPFQIVKNPRSVGKASEQLAGKVAEVKKNGRISLVLGGDHSLAIGSI
SGHARVHPDLGVIWVDAHTDINTPLTTTSGNLHGQPVSFLLKELKGKIPDVPGFSWVTPCISAKDIVYIGLRDVDPGEHILKTLGIKYFSMTEVDRLGIGKVMEETLS
YLLGRKKRPIHLSFDVDGLDPSFTPATGTPVVGGLTYREGLYITEEIYKTGLLSGLDIMEVNPSLGKTPEEVTRTVNTAVAITLACFGLAREGNHKPIDYLNPPK

>TRIBOLIUMARG
MFKKGSGVLWQLRKNYSTKIGIVGVPFEEGQGKVGVANGPEAMRKSNLIENIKSIHQEIDVHDYGDVCYTSLENVEVPNMRKYSDVAACNLQVSRTVEKILNDGRICL
TLGGDHSIGKFNNEYEKPKLQETFKAKNEKVCILWVDAHADLNTNKTSVSGNIHGMPLAILVKELADYWPYLPGMDWQKPVLPIRNVAYIGLRSVDSYERLIIEQFGI
TAYGMEDVENYGIHNIVNMALDRIDPHRMLSIHLSFDIDSLDALEAPSTGTAVRGGLTLREGIHLVEQIHKTGRLGAMDLVEVNPSIGSPKDVQKTVEAAVHLLMAAC
GYTRRGLIPRGPDGSPIRTIPSPV

>BOMBYXARG
MSQNTILKPLNRVGVIGVPFEKGQKKYGVSIAPAAVRAAGLIDELKEIDGLDVKDFGDIETSSCENNVNVNNMNHLPLVSACNKNLSERVSHVLKDGRIAVTVGGDHS
IGVGTVDGHYNVNEDMILIWVDAHADINTNKTSESGSVHGMPVALLVRELSDYWPYLPTMDWQVPRFSIKNLGYIGLRSVDKYERLAIEKYNVPTFTMEDVDLHGVEK
SITHLLKVLDPENRKPIHVSFDIDSLDALEAPSTGTPVRGGLTLREAIKLMEIIHATGRLRAIDLVEINPAIGNENDRKRTIEAGLCVLKAALGFSRKGSPPKGITDL
PIQTISNN

>CELEGANSARG
MKKSTQLARQVIRAIGCANGLAGRQLGCENAVEVIKASTYLAGVQTRLPLEWGKIIEEVNTGRHASAMSGVTQTCRQLAHETRQVIENKEELLVFGGDHSCAIGTWSG
VATAMRPVGDIGLIWVDAHMDAHTPDTSDTGNIHGMPVAHLLGFGDKTLVKIGDRLPKLLPHNLCMVGIRDYESAEQELLEKLGVRIFYAHEVEKRGIQDVMQEAQYL
VTRNTIGYGLSIDLDGFDVSYAPAVGTPSADGINALEFIKALLTIDLTKLIATEIVEFLPRFDDTQRTSEQLVSSLVEYIYKTKQFQINSVNEIAQRVSTSEQSQKIT
RAM

>PHUMANUSCORPORISARGN-TERMINALLYTRUNCATED
VSNCVSSALQDCRACLMMGGDHSLATGSIHGHISVAGPQNVGVIYIDAHADINTASSSLSGNIHGMTVAMLAEELRKFWPNSQVPGLECLPLGNIAFIGLRSVDPEET
VFLDENNVAAYTMEDVENYGLHKVLCEAIKRVDPNGNKSIHLSFDIDSLDPLEAPCTTVPVRGGLTLREGIKIGETLCRTKRLSVVDFVEVNPPLGSEIQRQTTLDAA
FHVLLSVFGYSRRG
