# Supplementary material for: Rapid functional diversification in the structurally conserved ELAV family of neuronal RNA binding proteins
Source: BMC Genomics. 2008 Aug 20;9:392. doi: 10.1186/1471-2164-9-392 (PMC2529313; doi:10.1186/1471-2164-9-392)
Supplement: Additional file 3 — Protein sequence comparison among 12 arginases from 11 metazoans. Arginase sequences alignment with legend. [file 1471-2164-9-392-S3.pdf]

```
H.sapiens (ARG2)    -RVHSILKKSVHSVAVIGAPFSQGQKRKGVEHGPAAIREAGLMKRLSSLG--CHLKDFGDLSFTP----VPKDDLYNNLIVNPRSVGLANQELAEVVSRA 93
H.sapiens (ARG1)    ------MSAKSRTIGIIGAPFSKGQPRGGVEEGPTVLRKAGLLEKLKEQE--CDVKDYGDLPFAD----IPNDSPFQ-IVKNPRSVGKASEQLAGKVAEV 87
N.vitripennis       -FFRKIGERQYSKVGIIGVPFDKGQRKAGVGEAPDIIRSAGLVNELQGLG--LNVHDYGNVQYET----ASIN---VDNMPNLGDVAACTQKLSEMVQQS 90
A.mellifora         -VIIRLGNRYYKKLGIIGVPFEKGQK--GVAQGPEAIRKAGLMKELELLG--LDVKDYGNILYKA----KNVAE--VDNMTHLGDVAGCTSKLSEQFQQI 88
B.mori              -MSQNTILKPLNRVGVIGVPFEKGQKKYGVSIAPAAVRAAGLIDELKEIDG-LDVKDFGDIETSS----CENNVN-VNNMNHLPLVSACNKNLSERVSHV 93
T.castaneum         -VLWQLRKNYSTKIGIVGVPFEEGQGKVGVANGPEAMRKSNLIENIKSIHQEIDVHDYGDVCYTS----LENVE--VPNMRKYSDVAACNLQVSRTVEKI 91
C.pipiens           AGLEKFKKINYEKIGIVGVPFEKGQRKKGVGLGPKAIREAGLIDSIQEISNKLDIRDYGDIRYEA----LNLQGRLAANMKKLEHVASCTRFLSERVTQV 96
A.aegypti           DGLEKFRKINYEKIGIVGVPFEKGQRKKGVGLGPKAIREAGLIDSIQEISTKLDIRDYGDIQYEA----LNLQGRLATNMKQLEHVASCTKLLSQRVTRV 96
A.gambiae           FATIKPKKINYERIGIVGVPFDKGQRKKGVGLGPKAIREAGLIDHIQEISPKLNIKDYGDIQYEA----LNFQGRKVGNMKKLEHVASCSRNLSHQVTEV 96
P.humanus corporis  --------------------------------------------------------------------------------------------VSNCVSSA  8
D.melanogaster      -TGSTAPREPEQSLGIIGVPFAKGQAKQGVELAPDLLRQSSLRQVLQSSHDGLVIRDYGNLQYAVDEPLLQQQRVHYHHIRNYADFMACNRALIEQVKLM 99
C.elegans           --MKKSTQLARQVIRAIGCANGLAGRQLGCENAVEVIKASTYLAGVQTRLP----LEWGKIIEEVN---------TGRHASAMSGVTQTCRQLAHETRQV 85
                                                                                                                :

H.sapiens (ARG2)    VSDGYSCVTLGGDHSLAIGTISGHARH----------CPDLCVVWVDAHADINTPLTTSSGNLHGQPVSFLLRELQDKVPQLPGFSWIKPCISSASIVYI 183
H.sapiens (ARG1)    KKNGRISLVLGGDHSLAIGSISGHARV----------HPDLGVIWVDAHTDINTPLTTTSGNLHGQPVSFLLKELKGKIPDVPGFSWVTPCISAKDIVYI 177
N.vitripennis       LRDGRRVLTIGGDHSIGIGSIDGHVKV----------DKDVAILWIDAHADLNTNKTSESGNVHGMPVALLASELADYWPYLPGMDWQQPIVSIRNVGYI 180
A.mellifora         LKKDRRILTLGGDHS-GIGTIDGHVKE----------KGDIALIWVDAHADLNTNKTSTTGLFHGMPVALLTSELADYWPHLPGMDWQKPMLSIRNVAYI 177
B.mori              LKDGRIAVTVGGDHSIGVGTVDGHYNV----------NEDMILIWVDAHADINTNKTSESGSVHGMPVALLVRELSDYWPYLPTMDWQVPRFSIKNLGYI 183
T.castaneum         LNDGRICLTLGGDHS--IGKFNNEYEKPKLQETFKAKNEKVCILWVDAHADLNTNKTSVSGNIHGMPLAILVKELADYWPYLPGMDWQKPVLPIRNVAYI 189
C.pipiens           LNEERLCLTLGGDHAIAIGSIDGHLKH----------CSDVAVIWVDAHADLNTNSTSPSGNIHGMPVALLAKELA-YWPYIPGMDWQEPIISIKNMVYI 185
A.aegypti           LNEDRLCLTLGGDHAIAIGSIDGHLKH----------CSDVGVIWVDAHADLNTNSTSPSGNIHGMPVALLAKELADYWPYIPGMDWQEPIISIKNMVYI 186
A.gambiae           LDDDRLCITLGGDHAIAIGSIDGHLHH----------SKDVAVIWVDAHADLNTNSTSPSGNIHGMPVALLARELCDYWPYIPGMDWQEPIISIKNLAYI 186
P.humanus corporis  LQDCRACLMMGGDHSLATGSIHGHISVAG--------PQNVGVIYIDAHADINTASSSLSGNIHGMTVAMLAEELRKFWPNSQVPGLE--CLPLGNIAFI  98
D.melanogaster      LVENTQFLAIGGDHAIGFGSVAGHLQH----------TPNLSLVWIDAHADINLHSTSQSGNIHGMPVSFLLEQLRNTWQHAGLQEIAPNCLPKDQLVYI 189
C.elegans           IENKEELLVFGGDHSCAIGTWSGVATAMR-------PVGDIGLIWVDAHMDAHTPDTSDTGNIHGMPVAHLLGFGDKTLVKIG---DRLPKLLPHNLCMV 175
                      .    : .****:   *.  .                .: ::::*** * :   :: :* .** .:: *                   .   .:  :*
                                                                                              X

H.sapiens (ARG2)    GLRDVDPPEHFILKNYDIQYFSMRDIDRLGIQKVMERTFDLLIGKRQRPIHLSFDIDAFDPTLAPATGTPVVGGLTYREGMYIAEEIHNTGLLSALDLVE 283
H.sapiens (ARG1)    GLRDVDPGEH-ILKTLGIKYFSMTEVDRLGIGKVMEETLSYLLGRKKRPIHLSFDVDGLDPSFTPATGTPVVGGLTYREGLYITEEIYKTGLLSGLDIME 276
N.vitripennis       GLRSVDRYERLVIEKFGITAFGMEDVERFGIHEVVNMALRKIDPDNNRSLHVSFDIDSLDPLEAPSTGTPVRGGLSLREGIHLMEDLYRTNRLNALDLVE 280
A.mellifora         GLREVDSYERLVIEKFGITAFGMEDIERYGIHDVTYMALSKIDPNDSRSIHVSFDIDSLDPLEAPCTGTPVRGGLSLREAIHLMEVLYRTKRLNALDIVE 277
B.mori              GLRSVDKYERLAIEKYNVPTFTMEDVDLHGVEKSITHLLKVLDPENRKPIHVSFDIDSLDALEAPSTGTPVRGGLTLREAIKLMEIIHATGRLRAIDLVE 283
T.castaneum         GLRSVDSYERLIIEQFGITAYGMEDVENYGIHNIVNMALDRIDPHRMLSIHLSFDIDSLDALEAPSTGTAVRGGLTLREGIHLVEQIHKTGRLGAMDLVE 289
C.pipiens           GLRSVDPYERLIIEKFGIHAFGMREVEQYGIHEVMRMALERVDPEGKKSLHVSYDIDSLDVLEAPSTGTSVRGGLTLREGIYIMEEAYNTGRLAAVDLVE 285
A.aegypti           GLRSVDPYERVIIEKFGIHAFGMREVEKYGINDVMKMALERIDPEGKKSLHVSYDIDSLDVLEAPSTGTGVRGGLTLREGIYIMEEAYNTGRLAAVDLVE 286
A.gambiae           GLRSVDPYERAIIEKFGINAFGMREVEKYGIREVMRMALERIDPNGERSLHVSYDIDSLDVLEAPSTGTSVRGGLTLREGIYIMEEAYGTGRLAAVDLVE 286
P.humanus corporis  GLRSVDPEETVFLDENNVAAYTMEDVENYGLHKVLCEAIKRVDPNGNKSIHLSFDIDSLDPLEAPCTTVPVRGGLTLREGIKIGETLCRTKRLSVVDFVE 198
D.melanogaster      GLRDIDPYEAFILNKVGIRYYAMDTIDRVGVPKIIEMTLDALNPQNK--IHVSFDIDALDSNVAPSTGTAVRGGLTLREGISIVEALRDTKRVQGVDLVE 287
C.elegans           GIRDYESAEQELLEKLGVRIFYAHEVEKRGIQDVMQEAQYLVT-RNTIGYGLSIDLDGFDVSYAPAVGTPSADGINALEFIKALLTIDLT-KLIATEIVE 273
                    :*. :  *   :.  .:  :    ::  *: .        :         :* *:*.:*   :*.. .   .*:.  * :        *  :   :::*

H.sapiens (ARG2)    VNPQLATSEEEAKTTANLAVDVIASSFGQTREGGHIVYDQLPTPSSPDESENQARVRI 341
H.sapiens (ARG1)    VNPSLGKTPEEVTRTVNTAVAITLACFGLAREGNHKPIDYLNPPK------------- 321
N.vitripennis       VNPRIGD-KKSVDFTVEAAIHIIQAGFGYSRRGLKVPEGITDLPLQTFR--------- 328
A.mellifora         INPYIGN-KYDVQLTIGAAIH-IQAGFGYSRRGLRVPEGVTDIPLPTVK--------- 325
B.mori              INPAIGN-ENDRKRTIEAGLCVLKAALGFSRKGS-PPKGITDLPIQTISNN------- 332
T.castaneum         VNPSIGS-PKDVQKTVEAAVHLLMAACGYTRRGL-IPRGPDGSPIRTIPSPV------ 341
C.pipiens           VNPSIGT-PDDVRKTLDAAIHLLVAACGHNRRGN-FADTLDIVGKKK----------- 330
A.aegypti           VNPSVGT-PEDVRKTLDAAIHLLVAACGHSRIGD-IADTIDLIKK------------- 329
A.gambiae           VNPAIGT-PEDVRRTVEAAIHLLVAACGHSRKGD-IADTLDLIQK------------- 329
P.humanus corporis  VNPPLGS-EIQRQTTLDAAFHVLLSVFGYSRRG------------------------- 230
D.melanogaster      INPKLGS-ERDVRTTVESGLEILKSMFGYRRSGRWSNIDTGILGSD------------ 332
C.elegans           FLPRFDDTQRTSEQLVSSLVEYIYKTKQFQINSVNEIAQRVSTSEQSQKITRAM---- 327
                    . * .              .            .
```

Protein sequence comparison among 12 arginases from 11 metazoans. "*" indicate that amino acids are identical in all the 27 sequences, ":" and "." respectively indicate conserved and semi-conserved substitutions. Grey boxes are used to identify amino acids encoded by exon junctions. When the splicing connects intact codons, two amino acids are boxed. The underlined sequences are arginase family signatures. The X below the sequences marks the boundary between regions of the *D. melanogaster* arginase encoded by sequences respectively upstream and downstream of intron 3. The boxed amino acids at this position correspond to codons where the first and second bases come from an exon and the third base from a downstream exon.
